# Neonatal venolymphatic malformation with spontaneous parotid duct rupture: first case report

**DOI:** 10.1093/omcr/omac060

**Published:** 2022-06-23

**Authors:** Darshanika C T Gamage, Bernard Deepal Wanniarachchi Jayamanne, Syed Faizan Quasim, Kosmos Kailidis, Anas Olabi

**Affiliations:** Department of Paediatrics, Furness General Hospital, UHMB Trust, Dalton Ln, Barrow-in-Furness, Cumbria LA14 4LF, UK; Department of Public Health, Faculty of Medicine, University of Kelaniya, Ragama 11010, Sri Lanka; Faculty of Health and Medicine, Lancaster University, Bailrigg LA1 4YW, UK; Department of Paediatrics, Furness General Hospital, UHMB Trust, Dalton Ln, Barrow-in-Furness, Cumbria LA14 4LF, UK; Department of Paediatrics, Furness General Hospital, UHMB Trust, Dalton Ln, Barrow-in-Furness, Cumbria LA14 4LF, UK; Department of Paediatrics, Furness General Hospital, UHMB Trust, Dalton Ln, Barrow-in-Furness, Cumbria LA14 4LF, UK

## Abstract

Disorders of salivary glands especially the parotid gland very rare among neonates and children other than cytomegaly and parotitis epidermica. Venolymphatic malformations are very rare in children. Such presentation around the parotid region yet to be reported. This case report describes a rare presentation of a neonatal venolymphatic malformation on the parotid duct. A 4-week-old termly delivered male infant referred to by a general practitioner bruising over the left buccal area for 1 day from non-consanguine healthy parents. On examination a bluish discoloration in the buccal mucosa over a firm mildly tender area without signs of inflammation was seen. Ultrasound examination of the lesion showed fluid and solid soft tissue suggestive of haematoma and magnetic resonance imaging scan confirmed the rupture of the parotid duct with venolymphatic malformation. The child has been referred to the vascular malformation clinic and plastic surgical clinic in a tertiary care hospital for follow-up.

## INTRODUCTION

Disease of the salivary gland is very rare in the neonates and children except in parotitis epidemica and cytomegaly [1]. Very rarely neonate and children can be present with suppurative parotitis due to staphylococcus infection where only 44 cases have been reported [2].

Vascular malformation of the children can appear from the antenatal period up to childhood and 60% of them are confined to the head and neck region [3]. There are several ways of classifying congenital vascular anomalies. The International Society for the Study of Vascular Anomalies (ISSVA) has described uniform classification. According to that classification congenital vascular anomaly is divided into two categories as vascular tumour and vascular malformation [4].

This is the first reported case of venolymphatic malformation of the parotid region in a neonate.

**Figure 1:**
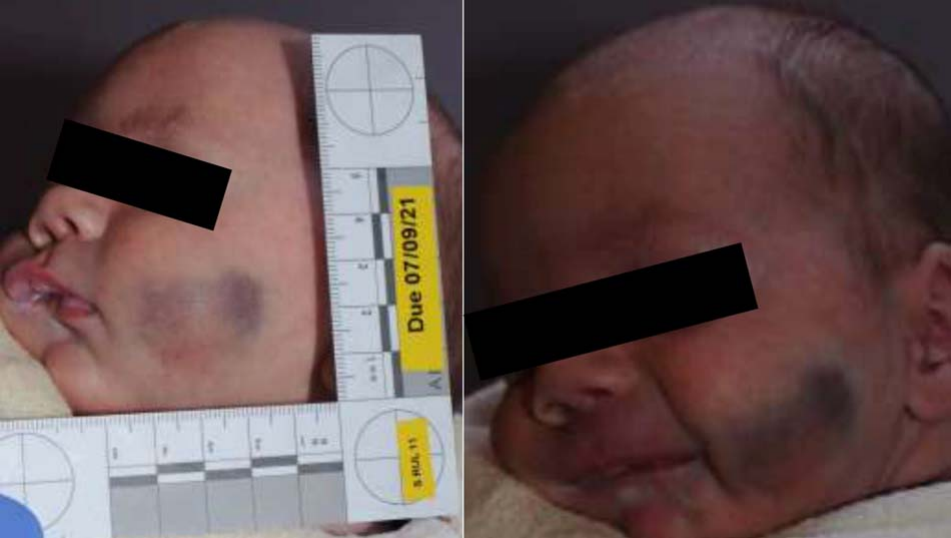
External appearance of the cheek. Bluish discoloration on the centre of the left buccal area measuring 3 cm horizontally and 2.5 cm in vertical plane.

## CASE REPORT

Four-week-old male infant was referred to the hospital by a general practitioner due to a swelling and bruising over the left buccal area for 1 day according to the history given by the mother. There was no history of trauma, coagulopathy, fever, irritability, poor feeding nor documentation of discolouration or patch during the first neonatal examination. The child was fed well, and the urine output and bowel frequency were normal.

This child was born as the second child to non-consanguine healthy parents with no family history of coagulopathies, after an uneventful antenatal period of 35 weeks by an emergency lower segment caesarean section due to foetal tachycardia. His birth weight was 2.62 kg. Then he was treated for suspected sepsis and neonatal hyperbilirubinemia at the special care baby unit and spent the first 2 days of life establishing feeding with nasogastric tube and formula top ups.

The clinical examination on admission showed bluish discoloration on the centre of the left buccal area measuring 3 cm horizontally and 2.5 cm in vertical plane (Fig. 1). Firm mild tender underlying swelling without features of trauma, active bleeding or signs of inflammation together with bluish discoloration was identified in the buccal mucosa. The rest of the physical examination, system examination and anthropometric measurements were normal.

**Figure 2:**
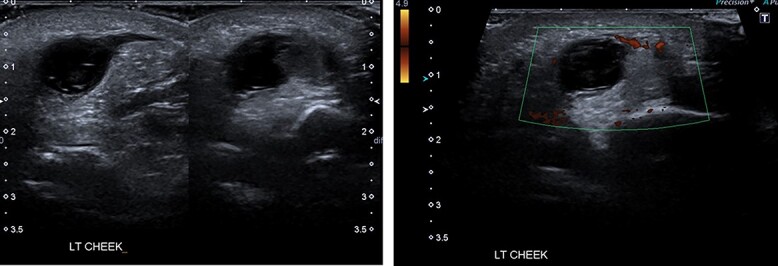
Ultrasound scan images of the lesion the left cheek, showing fluid and solid soft tissue suggestive of haematoma.

**Figure 3:**
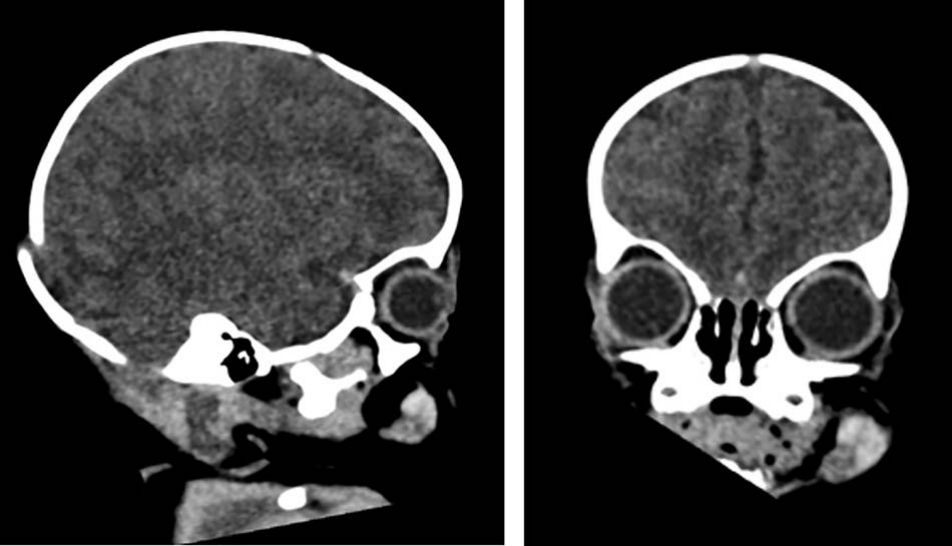
CT scan image (sagittal plane—left, coronal plane—right), showing 1–1.2-cm subcutaneous hyperdensity left buccal area with haematoma due to a possible pre-existing vascular malformation and no intracranial haemorrhages.

**Figure 4:**
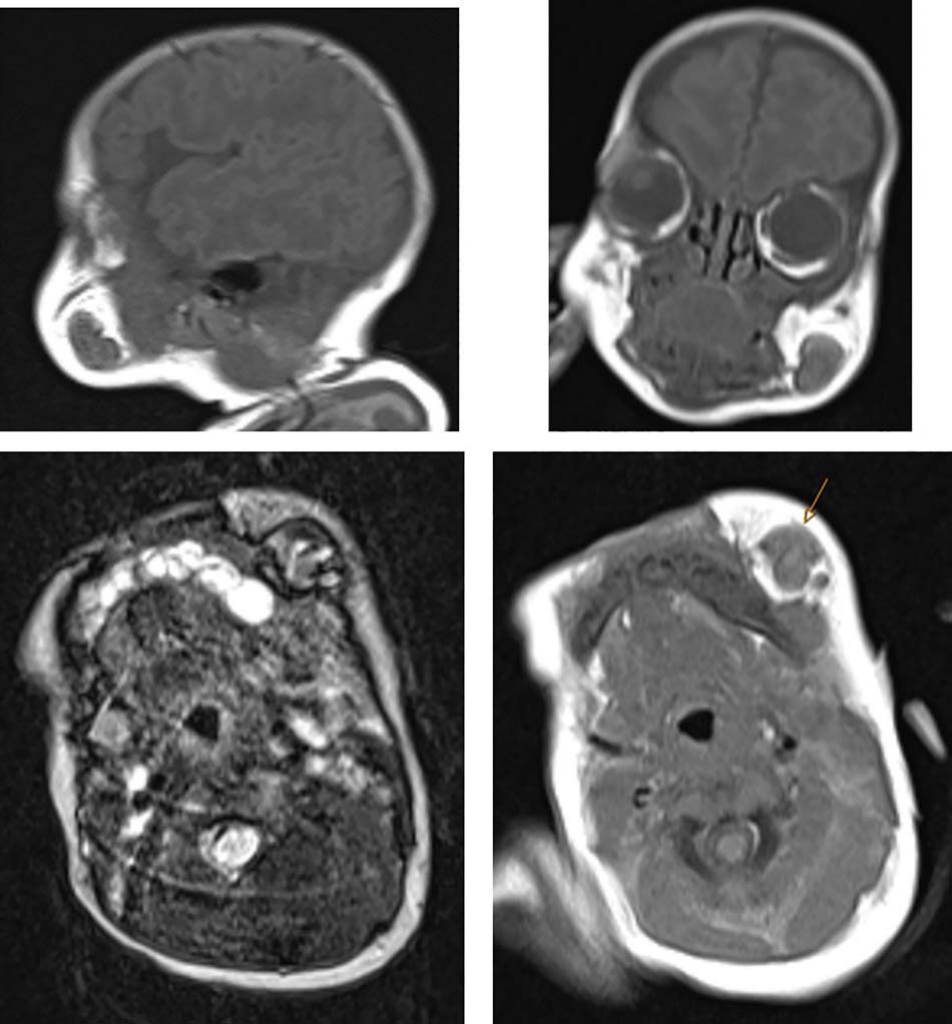
MRI showing the rupture of the parotid duct with venolymphatic malformation.

The laboratory investigations including full blood count, coagulation screen, inflammatory markers, renal profile, liver profile and bone profile were within normal limits [5]. Due to the nature of the presentation the possibility of non-accidental injury (NAI) was considered and investigations including skeletal survey, computed tomography (CT) head, fundoscopy and strategy multidisciplinary meeting took place. The ultrasound examination of the lesion showed fluid and solid soft tissue suggestive of haematoma (Fig. 2). CT scan of the head revealed that there is a 1–1.2-cm subcutaneous hyperdensity left buccal area with haematoma due to a possible pre-existing vascular malformation and no intracranial haemorrhages (Fig. 3). Magnetic resonance imaging (MRI) scan confirmed the rupture of the parotid duct with venolymphatic malformation (Fig. 4).

The child has been referred to the vascular malformation clinic and plastic surgical clinic in a tertiary care hospital (Alder Hay Children Hospital, Liverpool, UK) for further follow-up.

## DISCUSSION

Disorders of the salivary gland including parotid gland acute and chronic inflammation and infection [2] are exceedingly rare in neonates [1], and venolymphatic malformation around the parotic gland and presenting with bluish discolouration a few days after birth is rare. Most of the time vascular malformation is present as a birth mark.

Congenital vascular malformation arises because of structural malformations of vascular development [6], which appears from in utero to childhood but most of the time lesions are diagnosed at birth. Vascular malformation causes surrounding soft tissue abnormality that leads to functional and aesthetic impairment [6]. The diagnosis of the lesions and the treatments can be complicated, due to lack of signs and symptoms, lack of follow-up and limitation of specific diagnostic tests [7].

Majority of vascular malformation (~60%) in the children and neonate are confined to the head and neck region and it gives enormous psychological and physiological problems to both the child and the parents. All the congenital vascular anomalies are categorized into two main categories under vascular tumours and vascular malformation. Nomenclature of the vascular malformations depends on the predominant type of the vessel (lymphatic, arterial or venous) [6]. Venolymphatic malformation comes under a combined category of vascular malformation [8]. Vascular malformations are also categorized according to the haemodynamic characteristics under high–low and low flow. Venous, lymphatic and capillary malformations are categorized under low-flow malformation [9].

Vascular malformation around the parotid gland is an extremely infrequent entity that needs a specific diagnostic and management approach. Up to now, a few cases have been reported and all of them were adults [6].

When a nonmobile child, especially a neonate, present with bruises, NAI and coagulation disorders need be excluded. However, very rarely can neonate be presented with bruises due to the rupture of underlying vascular malformations. In this case, all the probable causes of bruises in neonate have been excluded, and the rupture of the parotid duct and venovascular malformation has been diagnosed by the MRI and repeated ultrasonography of the head and neck.

The plan of management for this child was watchful waiting for 3 months and then to be decided on further management depending on the nature of the residual lesion. According to Ana Paula Pinho Matos *et al.* (2021) [11], 15% (3/20) venolymphatic malformations has showed spontaneous resolution or stable and asymptomatic after 3–18-year follow-up study in Brazil. The spontaneous resolution rate for the cervico-facial macrocystic lymphatic malformations presenting at birth or later in life are 11.5% after 1-year follow-up study of 104 subjects [10], which is comparable with 13% [12] and 12.5% [13].

The medical options available are sclerotherapy, minimally invasive surgical resection, laser therapy and radiofrequency ablation. Systemic pharmacological treatment with sirolimus (non-calcineurin-inhibiting immunosuppressant) is considered for patients with diffuse and infiltrative lymphatic malformations refractory to previous therapy and/or when surgical approach is not feasible due to the risk of permanent iatrogenic damage (e.g. blindness). According to the single case report of successful improvement of a complex periorbital venolymphatic malformation with sirolimus and prednisolone (Kim D. *et al.* in 2015) combined therapy that was initially failed to respond to the sildenafil treatment and sclerotherapy may have a place in management.
